# Acoustic Coordinated Reset Neuromodulation in a Real Life Patient Population with Chronic Tonal Tinnitus

**DOI:** 10.1155/2015/569052

**Published:** 2015-10-19

**Authors:** Christian Hauptmann, Armin Ströbel, Mark Williams, Nitesh Patel, Hannes Wurzer, Tatjana von Stackelberg, Uwe Brinkmann, Berthold Langguth, Peter A. Tass

**Affiliations:** ^1^Institute of Neuroscience and Medicine-Neuromodulation (INM-7), Jülich Research Center, 52425 Jülich, Germany; ^2^CERES GmbH Evaluation & Research, 79539 Lörrach, Germany; ^3^The Tinnitus Clinic Inc., London W1G 6AX, UK; ^4^Tinnitus Zentrum Promenadeplatz, 80333 München, Germany; ^5^Ear, Nose and Throat (ENT) Center, 40667 Meerbusch, Germany; ^6^Ear, Nose and Throat (ENT) Clinic Hamm-Ahlen-Oelde, 59302 Oelde, Germany; ^7^Department of Psychiatry and Psychotherapy, University of Regensburg, 93053 Regensburg, Germany; ^8^Department of Neurosurgery, Stanford University, Stanford, CA 94305, USA; ^9^Department of Neuromodulation, University of Cologne, 50923 Cologne, Germany

## Abstract

*Purpose*. Primary tinnitus has a severe negative influence on the quality of life of a significant portion of the general population. Acoustic coordinated reset neuromodulation is designed to induce a long-lasting reduction of tinnitus symptoms. To test acoustic coordinated reset neuromodulation as a treatment for chronic, tonal tinnitus under real life conditions, an outpatient study “RESET Real Life” was commissioned by ANM GmbH. Herein we present the results of this study. *Methods*. In a prospective, open-label, nonrandomized, noncontrolled multicenter clinical study with 200 chronic tinnitus patients, tinnitus questionnaire TBF-12 and Global Clinical Improvement-Impression Scale (CGI-I7) are used to study the safety and efficacy of acoustic coordinated reset neuromodulation. 189 patients completed the last 12-month visit, 11 patients dropped out (8 because of nontreatment related reasons; 2 because tinnitus did not change; and 1 because tinnitus got louder). *Results*. Acoustic coordinated reset neuromodulation caused a statistically and clinically significant decrease in TBF-12 scores as well as in CGI-I7 after 12 months of therapy under real life conditions. There were no persistent adverse events reported that were related to the therapy. *Conclusion*. The field study “RESET Real Life” provides evidence for safety and efficacy of acoustic coordinated reset neuromodulation in a prospective, open-label, real life setting.

## 1. Introduction

The perception of sound in the absence of a corresponding sound source is the definition of primary tinnitus, which, in its chronic form, affects 10–15% of the general population in industrialized countries [1]. About 2% are severely impaired in their quality of life because of chronic tinnitus and rely on professional help [2, 3].

A growing body of evidence suggests that altered spectral power of neural signals, that is altered statistical distribution of power over frequency, as measured by EEG/MEG, is the neuronal fingerprint of primary tinnitus [4–10] and that the perception of tinnitus requires the involvement of a larger network of brain areas [11–14]. There are now several reports of oscillatory brain activity changes, recorded via EEG, in tinnitus patients that reveal a decrease in alpha wave power (10–14 Hz) within the primary auditory cortices [4, 9] and an increase in slow wave delta activity (1.5–4 Hz) [4, 9, 15] when compared to controls. Slow wave oscillations have been attributed to hyperpolarization of thalamic nuclei as a result of auditory deafferentation, which may enhance thalamocortical oscillations thus inducing pathological neural synchrony that has been proposed as the progenitor of tinnitus perception [16].

Various approaches have been investigated for the treatment of primary tinnitus [17] such as cognitive behavioral therapy [18], hearing aids [19], sound maskers [20], tinnitus retraining therapy [21], medication [22–24], hyperbaric oxygen therapy [25], acupuncture [26], and neuromodulation [27]. However, meta-analytic evidence has only been found for a beneficial effect from cognitive behavioral therapy on quality of life of tinnitus patients but not on tinnitus loudness [18].

Acoustic CR neuromodulation is a noninvasive acoustic stimulation therapy for primary tonal tinnitus, which was developed computationally [28–30]. The therapy is designed to counteract pathological neural synchrony by sustainably reducing the strength of synaptic connectivity between neurons within an affected cell population [8, 14]. In order to target the synchronized focus in the tonotopically organized auditory cortex, four acoustic tones are delivered with different frequencies centered around the characteristic frequency of the patient's tinnitus percept [8]. This approach aims to reduce pathologically enhanced neural synchrony within the primary auditory cortices which, in turn, results in a net decrease in effective connectivity across the global brain network involved in tinnitus perception [9, 14] along with a decrease of tinnitus-related abnormal cross-frequency coupling [31]. The tonotopically targeted stimulation tones are presented to the patient via a handheld tone generator (T30 CR neurostimulator), which utilizes earphones that are adapted from receiver-in-the-ear-canal (RIC) hearing aids. These RIC adapted earphones ensure that the patient's external auditory meatus is not occluded by the headphone receiver and enables a high degree of acoustic environmental transparency during stimulation tone presentation.

First evidence for acoustic CR neuromodulation as being an effective therapy for primary tonal tinnitus was provided by a randomized proof of concept trial: a statistically and clinically significant improvement in tinnitus questionnaire (TQ) and visual analogue scale (VAS) for loudness/annoyance scores was obtained [8, 32, 33]. Furthermore, the analysis of EEG recordings demonstrated a change in pathologically altered EEG power (i.e., *α*, *γ*, and *δ* band) towards normalization [8, 9].

To consolidate the results from the RESET proof of concept study in a larger patient population and in a real life outpatient setting, a second study, named RESET Real Life (RRL, ClinicalTrials.gov: NCT01435317) was conducted. The goal of the interventional multicenter RRL study was to collect data for the confirmation of efficacy and safety of twelve months of acoustic CR neuromodulation as a treatment of chronic tinnitus using the CE marked therapy system T30 CR. Tinnitus burden was assessed with the TBF-12 [34]; tinnitus loudness and annoyance were measured with numeric rating scales (NRS, ranging from 0 to 100) and clinical global improvement with the CGI-I7. In total, 200 patients were included in this prospective, open-label, nonrandomized, noncontrolled multicenter study at 23 study sites. Herein, we present the final data after 12 months of therapy.

## 2. Materials

200 patients were enrolled in this multicenter clinical study on Acoustic CR neuromodulation between November 2011 and May 2012 in 23 study centers run by ENT specialists located in Germany. Inclusion criteria were symptomatic primary tonal chronic tinnitus (≥3 months), <60 dB hearing loss for all tested frequencies (125 Hz–8 kHz), and men and women ≥ 18 years old. Patients were not included in the study if they were found to be suffering from serious neurologic, psychiatric, or otological disease, objective tinnitus (e.g., tinnitus caused by muscle movements, vascular noise, and other somatosounds), Meniere's disease, and tinnitus triggered by craniomandibular disorders.

Regular visits took place 0.5, 1, 2, 3, 6, 9, and 12 months after treatment start. The mean age of the patients was 50.6 years at study start, and 76.3% of the patients were male (Table 1). 62.1% of patients had undergone two or more tinnitus treatments prior to acoustic CR neuromodulation without significant relief. Among them 18.7% of patients had undergone more than 5 previous tinnitus treatments. Only 15.2% of patients were treated with Acoustic CR neurostimulation as a first line therapy. Most of the patients (68.2%) suffered from bilateral tinnitus (see Table 1).

Subjects were asked what they believe was responsible for inducing their tinnitus. We are aware about the limited reliability of subjective causal attributions to tinnitus onset. Nevertheless, the answer to this question provides some orientation about individual etiologic factors [35, 36]. 19 (9.5%) patients responded that it is noise trauma, 22 (10.9%) hearing problems, 95 (47.3%) stress, and 65 (32.8%) other reasons (multiple responses were allowed). The tinnitus severity (based on the initial TBF-12 measurement) was* slight (no handicap)* for 31.8% (TBF-12 0–8 pts),* mild* for 34.4% (TBF-12 9–12 pts),* moderate* for 24.1% (TBF-12 13–17 pts), and* severe* for 9.7% of the patients (TBF-12 18–24 pts). Tinnitus duration was less than six month for 2.0%, between six months and four years for 32.0%, between four years and ten years for 29.4%, and more than ten years for 36.6% of the patients. 43.1%/11.3% showed no hearing impairment (averaged/maximal hearing impairment ≤20 dBHL), 52.8%/19.5% a mild hearing impairment (averaged/maximal hearing impairment between 20 dBHL and 40 dBHL), 4.1%/42.0% a moderate hearing impairment (averaged/maximal hearing impairment between 40 dBHL and 60 dBHL), and 0.0%/27.2% a severe hearing impairment (averaged/maximal hearing impairment >60 dBHL).

The treatment with acoustic CR neuromodulation required regular visits to ENT clinics. At the first visit, a thorough pitch matching process was carried out to determine the tinnitus frequency. Based on the evaluated tinnitus frequency *f*_*T*_, four stimulation tones were defined, two below and two above the individual tinnitus frequency (frequency range: [0.76*f*_*T*_ : 1.4*f*_*T*_]). The amplitudes of the stimulation tones were adjusted in order to ensure that all tones were comfortably audible, at the same subjective loudness level, and slightly above the patient's hearing threshold.

**Figure 1:**
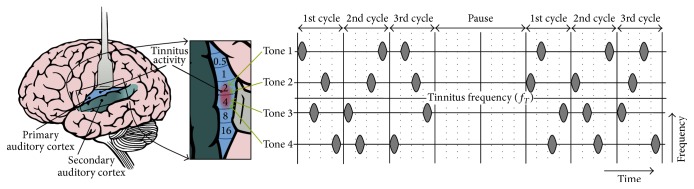
Stimulation signal of acoustic CR neuromodulation.

With this information (frequency and amplitude of stimulation tones), the handheld T30 CR device was programmed with a randomized tone sequence which consisted of the repetitive application of the four stimulation tones with a repetition rate of 1.5 Hz. Short pauses within the stimulation signal (CycOn = 3, CycOff = 2) are utilized in order to enhance the process of unlearning pathological tinnitus activity [37]. The stimulation pattern containing the four stimulation frequencies is designed to induce a phase reset of abnormal delta oscillations at different locations within the tonotopically organized auditory cortex (see Figure 1).

The therapy was applied using the T30 CR neurostimulator, which consists of a programmable, battery-powered device combined with a customized, open fit earphone that utilizes a receiver-in-the-ear-canal (RIC) technology. The prescribing clinician uses propriety software to program the T30 CR neurostimulator. The patients were asked to use their T30 CR neurostimulator device every day for 4–6 hours, applying the therapy signals either continuously or splitting up the stimulation time into sessions not shorter than one hour. Visits to the ENT clinic took place at the beginning of the therapy and then 0.5, 1, 2, 3, 6, 9, and 12 months after beginning of the therapy. At each visit to the ENT clinic, the tinnitus tone was measured by a thorough pitch matching process and the device was reprogrammed when the tinnitus tone was found to have changed.

The primary outcome measure of the study is the analysis of changes in tinnitus severity, measured by the German version of tinnitus handicap inventory (TBF-12,* Tinnitus-Beeinträchtigungs-Fragebogen*) or improvement of the Clinical Global Impression-Improvement Scale (CGI-I7). Improvement was defined as a statistically significant (*P* < 0.05) reduction of scores comparing baseline values to end of treatment (visit after 12 months). All scores were obtained in the off-stimulation situation.

The TBF-12 tinnitus handicap inventory consists of 12 questions leading to a total score of 24 points (worst result). Scores were categorized depending on the score recorded:* slight (no handicap)* (0–8 pts),* mild handicap* (9–12 pts),* moderate handicap* (13–17 pts), and* severe handicap* (18–24 pts) [38]. CGI-I7 consists of the seven categories:* very much improved*,* much improved*,* slightly improved*,* no change*,* slightly worse*,* much worse*, and* very much worse,* expressed by the numbers 1 to 7 (1 equals the* very much improvement category*).

For the final analysis, outcome measurements for the full 12 months of therapy were analyzed. For the statistical analyses the *t*-test was utilized to evaluate the TBF-12 and the sign test to evaluate the CGI-I7. This final analysis reports the results of the primary (TBF-12, CGI-17) and secondary endpoints (numeric rating scale (NRS) for tinnitus loudness and annoyance). Additionally, the patients were asked to report their device usage pattern to allow for the assessment of compliance.

The *t*-tests were applied as paired, one sided, equal variances, equal sample size tests to TBF-12 (total score), NRS loudness, and NRS annoyance. Scores before and after treatment were compared. Null hypothesis was the acquisition of equal mean scores before and after treatment. Alternative hypothesis was the acquisition of smaller means after treatment. The sign test was applied to CGI. CGI was grouped in 2 categories “improvement” and “equal/worse.” Null hypothesis was the acquisition of same number of patients in “improvement” and “equal/worse” category. Alternative hypothesis was more patients in the “improvement” category. Significance level was set to 0.05. No multiple testing corrections were applied. Missing values in the data were treated as missing for the analysis. Data from drop-out patients was treated by LOCF (last observation carried forward). To determine the predictors for therapy success receiver operating characteristics (ROC), curves were calculated using the 12-month data. TBF-12 total score, NRS loudness, and NRS annoyance were used as potential predictors and therapy success was defined as CGI-I7 ≤ 3.

The study was performed in accordance with good clinical practice guidelines and local ethics committees. All participating patients gave their written informed consent. Independent external clinical research associates and a clinical physician monitored the safety of the study. Data analysis was performed by an external contract research organization.

## 3. Results

189 patients finished the 12-month treatment and 11 patients dropped out for different reasons: eight stated nontreatment related reasons; two stated that their tinnitus did not change; and one stated that his tinnitus got louder.

The treatment was well tolerated and no serious adverse events (AE) were reported. An adverse event was defined as any untoward medical occurrence. Technical and handling problems were also documented as AE for this study. 89 product-related AEs were reported. Of these 89 product-related AEs, 40 were device related (i.e., technical and handling problems, rapidly solved). The other 49 AEs are considered to be therapy related. These were an additional atonal noise (15), additional tinnitus tone (3), increase in tinnitus burden (2), increase in loudness (13), tinnitus frequency shift (1), headaches (2), anxiety (1), tinnitus frequency increased to >10 kHz (2), discomfort (7), itching of ear canals (2), and otalgia (1). All adverse events were recorded as being temporary with no permanent or sustainable features.

**Figure 2:**
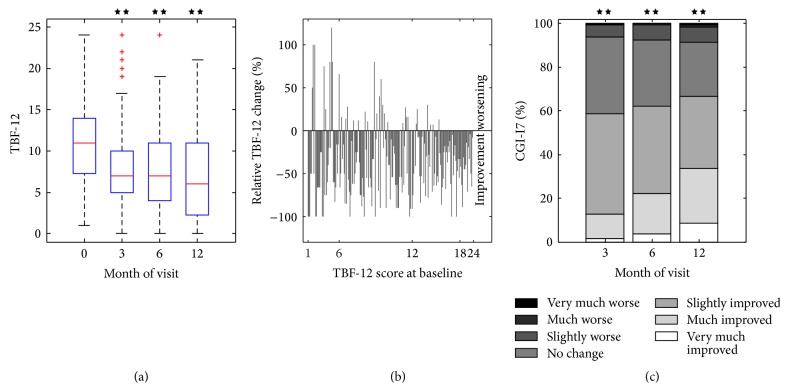
(a) TBF-12 scores at baseline and after 3, 6 and 12 months of treatment. (b) Relative TBF-12 change for the individual patients. The position of the bar on the *x*-axis indicates the TBF-12 score at baseline. (c) Distribution of the CGI-I7 scores after 3, 6 and 12 months of treatment. Stars indicate statistically significant results (^★★^ *P* < 0.01).

After 12 months, TBF-12 (total score) showed a mean reduction of 4.1 points (−37.9%) compared to baseline (*P* < 0.01, df = 191, *t* = −12.3, Table 2, Figures 2(a) and 2(b)): mean TBF-12 score at baseline was 10.8 points and after 3 (6/12) months 7.9 (7.5/6.7) points. After 12 months, the number of patients within the TBF-12 categories* moderate* and* severe handicap* decreased from 33.8% to 13.9%, while the number of patients within the category* slight (no handicap)* increased from 31.8% to 64.1%. The TBF-12 based effect size of the treatment is 0.89, which corresponds to a large effect size.

At the visit after 12 months the results of CGI-I7 revealed that 131 (66.9%) patients reported an improvement of their tinnitus, that is, CGI-I7 categories 1, 2, or 3 (*P* < 0.01, *k* = 131, *N* = 196, Table 2, Figure 2(c)), 24.5% felt no change (category 4), and 8.6% reported feeling that their tinnitus had become worse (categories 5, 6, and 7) (Table 2). After 3 months of treatment 58.59% (*P* < 0.01, *k* = 116, *N* = 198) of the patients reported an improvement of their tinnitus.

After 12 months of treatment the loudness of tinnitus, as obtained by a numeric rating scale (0–100), was reduced by 11.1 points (18.9%) compared to baseline (*P* < 0.01, df = 194, *t* = −4.53, Table 3): mean NRS loudness at baseline was 58.6 points and after 3 (6/12) months 53.7 (51.0/47.5) points. Tinnitus related annoyance (also obtained by a numeric rating scale (0–100)) was reduced by 14.7 points (25.2%) after 12 months of treatment as compared to baseline (*P* < 0.01, df = 197, *t* = −3,14, Table 3). When asked if they are free of tinnitus, after 12 months of treatment 54.4% of the patients reported either that they are tinnitus-free (4.1%) or that tinnitus has no negative influence on their life any more (50.3%).

In the course of the treatment the tinnitus pitch changed. In average a reduction of tinnitus pitch was observed (−11.2% after 3 months (*P* < 0.05, df = 361, *t* = −2.44) and −15.6% after 12 months of treatment (*P* < 0.01, df = 359, *t* = −3.20)). 55.6% of the patients showed a reduction of tinnitus frequency of more than 10%, 33.1% of the patient showed an increase of tinnitus frequency of more than 10%, and for the remaining 11.3% of patients the tinnitus frequency changed less than 10%. 80.0%/58.7% of patients with a reduction of tinnitus frequency >10% showed an improvement of TBF-12/NRS loudness, while this correlation is not significant (chi-squared test). These tinnitus pitch changes imply an adjustment of the therapy tones, which was done during the regular visits.

Based on the TBF-12 scores at baseline the patients were divided into four separate subgroups relating to tinnitus severity* slight (no handicap), mild, moderate*, and* severe*. At the end of the study, these scores were recorded as being reduced by 34.1%, 36.4%, 39.0%, and 41.5%, respectively, compared to the scores recorded at the beginning of therapy. The NRS loudness was reduced for these same subgroups by 20.7%, 17.2%, 17.6%, and 24.7% while the NRS annoyance was reduced by 18.5%, 21.9%, 32.2%, and 32.7%, respectively.

On average, the stimulation device was used by patients for five hours per day. Compliance was 87% at the beginning and fell to 73% after 12 months. “Compliance” was self-expressed by the patients and was defined as at least 4-hour daily stimulation. If the stimulation was split, then each stimulation block should be at least 1 hour long. We calculated receiver operating characteristics (ROC) curves to identify predictors for therapy success (therapy success was defined as CGI ≤ 3). Based on the 12-month data, the area under the curve (AUC) was as follows: TBF-12 total score: 0.73, NRS loudness: 0.82, and NRS annoyance: 0.83.

## 4. Discussion

This prospective, open-label, nonrandomized, noncontrolled multicenter clinical study with 200 chronic tinnitus patients demonstrates safety and good applicability, that is, good patient compliance and low drop-out rate, of acoustic CR Neuromodulation. 189 patients finished the 12-month treatment, which demonstrates a good patient adherence.

The applied treatment, acoustic CR neuromodulation, consists of a particular temporal pattern of stimulation tones intending the induction of local desynchronization of pathologically enhanced neuronal synchronization, which is the neuronal correlate of the tinnitus symptoms. By inducing desynchronization, which also affects limbic structures associated with the emotional perception of tinnitus [8, 9, 14], the stimulation signals start the process of unlearning the pathological signal, with the aim of resulting in a long-term reduction of the tinnitus symptoms.

Analysis of the results of this multicenter clinical study demonstrates significant results in both primary endpoints. Both the TBF-12 and CGI-I7 results are statistically and clinically significant after 12 months of treatment [39]. The initial tinnitus severity had only moderate effects on the treatment effect. Furthermore, this study serves to demonstrate the safety of acoustic CR neuromodulation, since, of all device-related adverse events, none was serious (i.e., life threatening or caused a hospitalization of the patient or disablement, etc.).

The final results of the RRL study, including data from 200 patients, support the results of the original RESET study: similar results were obtained within the larger patient population under “real life” conditions. While in the RESET study after 3 months of treatment (group 1, same treatment as in RRL) a change of −28.8% was obtained for the tinnitus questionnaire (TQ), the current study revealed a change of −27.3% in TBF-12 scores after a similar duration (visit after 3 months) and −37.9% after 12 months. This indicates that a continuation of the treatment beyond the initial 3 months can be very beneficial for the patient. Continuous improvement over the whole duration of the study was also found for tinnitus loudness and annoyance and may suggest that a treatment duration beyond 12 months may further increase treatment efficacy.

The authors are aware of the limitations of this study, which has been designed as an open study without a control group. Thus, it is not possible to reach a final conclusion with regards to what extent the observed effects are unspecific and to what extent they actually represent the specific effects of CR neurostimulation. However, the ongoing improvement of patients over 12 months and their relatively high resistance to previous treatments make placebo effects highly unlikely. A spontaneous recovery is unlikely as well, given the relative long tinnitus duration of most patients. In 88.1% of the patients, a positive treatment effect over the first 3 months (i.e., CGI-I7 ≤ 3 at month 3) correlated with a positive treatment outcome after 12 months of therapy (i.e., CGI-I7 ≤ 3 at month 12). 58.6% of the patients recorded a positive effect after 3 months of treatment and 66.5% after 12 months. TBF-12 improvement was seen to augment in this CGI-I7-based responder population from −31.9% (3 months) to −50.8% (12 months), NRS annoyance changed by −21.6% (3 months) and −35.6% (12 months), and NRS loudness changed by −16.7% (3 months) and −31.7% (12 months), while the CGI-I7-based nonresponders showed only moderate changes (TBF-12: −21.3% and −20.6%, NRS annoyance: +0.1% and −5.4%, and NRS loudness: +3.9% and −0.3% at 3 months and at 12 months, resp.). NRS annoyance resulted in the highest value in the ROC test, which indicates that it is a good metric for therapy success. Therefore, CGI-I7 combined with TBF-12 and NRS annoyance seems to be a reliable and easy to handle set of questionnaires and metrics, which can be used in ENT outpatient settings as indicators of treatment effects.

Our data (−37.9% mean change in TBF-12) can be compared with recent results on effects of standard tinnitus care and cognitive behavior therapy (CBT) [40]. Standard care (hearing aid or tinnitus masker, *n* = 161, 8-month treatment) resulted in a change of tinnitus handicap inventory (THI) and tinnitus questionnaire (TQ) by −11.9% and −13.3%, respectively, while specialized care (CBT, *n* = 175, 8 months treatment) resulted in a change of THI and TQ by −26.5% and −26.2%, respectively.

Thus, in summary, the RRL study reveals that acoustic CR neuromodulation, when applied for 12 months and used 4–6 hours per day in patients suffering from primary and tonal or tone-like tinnitus, is a safe and feasible technique and exerts encouraging effects on tinnitus loudness and severity.

## Figures and Tables

**Table 1:** Demographic data of study population.

Gender	
male	151 (76.3%)
female	47 (23.7%)
Age	
mean (std.)	50.6 yrs (10.4)
Perception of tinnitus	
unilateral	63 (31.8%)
bilateral	135 (68.2%)
Pretreatment	
none	30 (15.2%)
one	45 (22.7%)
≥two	123 (62.1%)
Cause of tinnitus	
noise trauma	19 (9.5%)
hearing problems	22 (10.9%)
stress	95 (47.3%)
Tinnitus severity	
slight (no handicap)	62 (31.8%)
mild	67 (34.4%)
moderate	47 (24.1%)
severe	19 (9.7%)
Tinnitus duration	
<6 months	4 (2.0%)
6 months to 4 years	63 (32.0%)
4 years to 10 years	58 (29.4%)
>10 years	72 (36.6%)
Hearing impairment 250 Hz–8.000 Hz (averaged/maximal)	
≤20 dBHL	84 (43.1%)/22 (11.3%)
20 dBHL–40 dBHL	103 (52.8%)/38 (19.5%)
40 dBHL–60 dBHL	8 (4.1%)/82 (42.0%)
>60 dBHL	0 (0.0%)/53 (27.2%)

The information concerning the cause of tinnitus is based on a self-assessment of the patient and the tinnitus severity is based on the TBF-12 scores (missing values are not taken into account, the total number of subjects varies between variables, and percentages are calculated without taking missing values into account). Hearing impairment is listed with two values: the averaged hearing impairment (in dBHL as measured by pure tone audiometry as described in DIN EN ISO 8253-1 within the range from 250 Hz to 8.000 Hz), for example, averaged over all frequencies of the audiogram, and the maximal hearing impairment, for example, the maximal impairment observed at one frequency.

**(a):** 

Variable	Visit [month]	*N*	Mean	SD	Delta	*P* value
TBF-12 Score	0	195	10.8	5.0	n.a.	n.a.
	3	193	7.9	4.7	−27.3%	<0.01
	6	193	7.5	4.7	−30.8%	<0.01
	12	195	6.7	5.0	−37.9%	<0.01

**(b):** 

CGI-I7	Patients		
(after 12 months, *N* = 196)	Number	Relative number	*P* value
Very much improved (1)	17	8.7%	<0.01
Much improved (2)	49	25%	
Slightly improved (3)	65	33.2%	
No change (4)	48	24.5%	
Slightly worse (5)	14	7.1%	
Much worse (6)	2	1%	
Very much worse (7)	1	0.5%	

**Table 3:** NRS scales for loudness and annoyance.

Variable	Visit [month]	*N*	Mean	SD	Delta	*P* value
NRS loudness	0	197	58.6	21.9	n. a.	n. a.
	3	198	53.7	20.6	−8.4%	<0.05
	6	197	51.0	21.5	−13.0%	<0.01
	12	196	47.5	24.9	−18.9%	<0.01

NRS annoyance	0	198	58.3	25.2	n. a.	n. a.
	3	198	50.9	21.8	−12.7%	<0.01
	6	196	47.7	23.1	−18.2%	<0.01
	12	196	43.6	25.7	−25.2%	<0.01
